# Subgroup-specific roles of primary cilia in medulloblastoma

**DOI:** 10.1101/gad.349856.122

**Published:** 2022-06-01

**Authors:** Silvia Marino

**Affiliations:** Blizard Institute, Barts and The London School of Medicine and Dentistry, Queen Mary University of London, London E1 2AT, United Kingdom

**Keywords:** cell cycle, cilia, medulloblastoma, translation, WNT

## Abstract

Here I discuss the study in this issue of *Genes & Development* by Youn et al. (pp. 737–751), which describes defined and diverse roles of primary cilia in molecularly distinct medulloblastoma subgroups, highlighting once again the importance of designing subgroup-specific therapeutic approaches for this tumor.

## Abstract (precis)

In this Outlook, Marino discusses a study in this issue by Youn et al. that describes defined and diverse roles of primary cilia in molecularly distinct medulloblastoma subgroups, highlighting once again the importance of designing subgroup-specific therapeutic approaches for this tumor.

Medulloblastoma, the most common malignant primary brain tumor in children, is a paradigm of deregulated developmental mechanisms leading to tumor formation (Marino 2005). It is classified into four subgroups (SHH, WNT, G3, and G4), each of which is further subdivided into subtypes. The pathogenetic links between these tumors and defined progenitor cells of the developing brain, as well as the deregulation of fundamental signaling pathways governing essential properties of these progenitors, are well established (for review, see Marino and Gilbertson 2021).

In the brain, primary cilia—microtubule-based cellular structures anchored to a basal body that serves as a template for the assembly of the ciliary microtubule (Larsen et al. 2013)—are critical to its development. They protrude from the surface of cells, sense multiple signals, and transduce essential signaling pathways, including the key developmental pathways sonic hedgehog (SHH) and WNT. For example, cilia play a key role in SHH-driven forebrain patterning, including interneuron migration; in cerebellar development, particularly expansion of cerebellar progenitors; and in hippocampal neurogenesis (for review, see Park et al. 2019). WNT-mediated dendritic refinement and synapse formation in adult-born dentate granule cells in the hippocampus are also processes mediated by cilia (Kumamoto et al. 2012). Primary cilia are involved in the pathogenesis of various brain tumors, including medulloblastoma (Han et al. 2009), choroid plexus tumors (Li et al. 2016), and glioblastoma (Goranci-Buzhala et al. 2021); however, the mechanistic underpinnings of their roles are just beginning to be unraveled.

The presence of primary cilia is known to be associated with the molecular subgroups of human medulloblastoma; they are found in SHH and WNT subgroups but not in G3 and G4 subgroups, a finding that is well mirrored by mouse models of these subgroups (Han et al. 2009; Zhao et al. 2017). In particular, the seminal work of Han et al. (2009) showed that primary cilia are required for or inhibit SHH-driven medulloblastoma formation, depending on the initiating oncogenic event—constitutively active SMO or GLI2, respectively. This work highlighted for the first time the heterogeneity of biological functions mediated by cilia in these tumors and their dependency on the cellular and molecular contexts. This could have important clinical implications given that cilium loss has been shown to confer SMO inhibitor resistance in SHH medulloblastoma (Zhao et al. 2017).

The elegant study of Youn et al. (2022) reported in this issue of *Genes & Development* has now significantly expanded our understanding of the multifaceted role of cilia in medulloblastoma pathogenesis. The investigators show that in mice, primary cilia are required to enhance the proliferation and survival of ectopically accumulated dorsal brainstem cells; i.e., the cells of origin of WNT medulloblastoma. Moreover, tumors that formed in mice lacking cilia showed decreased proliferation and increased cell death compared with WNT medulloblastomas arising in control mice. Fully transformed WNT medulloblastoma cells also require primary cilia for continuous proliferation and optimal survival. Mechanistically, cilia control β-catenin synthesis rate, as assessed by cycloheximide/proteasome inhibitor treatments in cell lines, via promoting nuclear accumulation of ELAVL1. Consequently, cilium loss increased stress granules containing β-catenin mRNA, the translation of which became disrupted. Hence, primary cilia promote WNT medulloblastoma by facilitating the synthesis of β-catenin, the major oncogenic driver in 90% of WNT medulloblastomas.

Interestingly, the investigators show a very different scenario in G3 medulloblastoma. Here, cilia are present in the progenitor cells, giving rise to G3 medulloblastomas; however, they are progressively lost during neoplastic transformation, and this significantly shortened the lives of mice with tumors. In particular, proliferation was increased via altered cell cycle dynamics, as assessed by double-thymidine analog labeling; the G2 phase was affected via abnormal CDK1 activation in the absence of cilia, which shortened the S phase and triggered premature G2/M transition. DNA damage and genome instability contributed to tumorigenesis in this scenario.

This study has expanded the repertoire of ciliary functions and further highlighted their context dependency. More work will be required to assess whether and how these mechanisms can be exploited therapeutically. However, this study emphasizes once again the importance of a detailed molecular understanding of the characteristics of medulloblastoma subgroups if novel patient-tailored more effective and less toxic therapies are to be developed for these tumors.
